# Physical Activity Counseling in UK General Practice: Understanding the Persistent Implementation Gap

**DOI:** 10.7759/cureus.117830

**Published:** 2026-10-12

**Authors:** Mustafa Al-Gburi

**Affiliations:** 1 General Practice, Acklam Medical Centre, Middlesbrough, GBR

**Keywords:** behavior change, family medicine, general practice, implementation, moving medicine, physical activity, prevention, primary care, public health, sport and exercise medicine

## Abstract

Physical activity (PA) counseling is a high-value intervention endorsed by national guidelines, yet its delivery in UK general practice remains inconsistent despite strong evidence supporting its effectiveness. Multiple studies demonstrate that brief PA interventions in primary care increase activity levels and improve clinical outcomes across a range of long-term conditions. However, uptake and documentation of PA discussions remain low. This editorial examines the structural, digital, behavioral, and organizational barriers that contribute to the persistent implementation gap in PA counseling. Evidence suggests that while clinicians recognize the importance of PA promotion, real-world delivery is undermined by time pressure, fragmented digital tools, limited confidence in behavior change techniques, and insufficient signposting options. Resources such as Moving Medicine provide structured frameworks, but their lack of integration into electronic health record systems limits routine adoption. Organizational drivers, including the absence of incentives and inconsistent coding practices, further restrict implementation. Addressing these challenges requires a shift from educational tools to system-embedded support, including electronic health record-integrated prompts, unified signposting pathways, incentive alignment, and improved training. Strengthening these elements is essential if PA counseling is to become a reliable component of routine primary care. This editorial synthesizes key barriers identified in recent literature and outlines strategies for enhancing implementation in UK general practice.

## Editorial

Physical activity (PA) is one of the most important modifiable determinants of health. Regular PA reduces the risk of cardiovascular disease, type 2 diabetes, several cancers, depression, and premature mortality while supporting healthy aging across the life course [1]. The UK Chief Medical Officers’ guidelines emphasize that even relatively small increases in activity among the least active can produce meaningful health benefits [1]. Despite this, consistent delivery of PA counseling in UK general practice remains limited. The gap between what national guidance recommends and what occurs in everyday consultations appears to reflect practical and organizational constraints rather than simply a lack of clinician interest.

Brief PA interventions delivered opportunistically in primary care can increase activity levels in adults. Orrow et al.’s systematic review and meta-analysis demonstrated that primary care-based interventions can produce increases in PA at 12 months [2]. NICE guidance on brief PA advice similarly recommends identifying adults who are inactive and providing brief, structured advice tailored to the individual [3]. General practice is therefore well positioned to promote PA because of its repeated contact with people living with long-term conditions and those at increased risk of future disease. However, translating these recommendations into routine practice requires systems that make PA counseling feasible within already complex consultations.

Clinician attitudes illustrate this challenge. Lowe et al. found that GPs generally regard PA promotion as important, but confidence and routine delivery are considerably less consistent [4]. Time pressure, competing clinical priorities, uncertainty about how best to deliver PA advice, and the complexity of consultations involving multimorbidity can all make preventive discussions difficult to prioritize. Consultation-level research provides a similar picture. Grice et al., analyzing video-recorded general practice consultations, found that although PA was relevant to many consultations, explicit discussions occurred much less frequently [5]. When PA was discussed, conversations were often brief and offered limited opportunity to explore individual preferences or practical options [5]. Together, these findings suggest that the implementation gap is not simply a question of whether clinicians value PA, but whether routine primary care provides sufficient opportunity and support to address it consistently.

Digital infrastructure is particularly relevant. General practice consultations must accommodate presenting problems, long-term condition management, medication review, documentation, preventive care, and safety netting within limited time. PA counseling can consequently be displaced unless it forms part of established clinical workflows. Resources such as Moving Medicine provide structured, condition-specific approaches to discussing PA, but requiring clinicians to move between external resources and the electronic health record may add friction during time-limited consultations.

Importantly, existing electronic templates already provide part of the infrastructure needed to address this problem. Ardens resources for EMIS and SystmOne support the Quality and Outcomes Framework (QOF) and long-term condition reviews through structured templates, clinical coding, and prompts for outstanding work. PA is also incorporated into some existing workflows; for example, Ardens NHS Health Check resources support recording of PA through the General Practice Physical Activity Questionnaire [6]. However, PA assessment and counseling are not consistently embedded as routine elements across all chronic disease reviews. The opportunity may therefore be less about creating another standalone resource and more about using existing consultation infrastructure to make PA assessment, brief advice, and appropriate coding more visible and consistent. Embedding proportionate PA prompts within relevant long-term condition templates could reduce reliance on clinicians remembering to initiate the discussion independently and help normalize PA alongside other routinely assessed risk factors.

Local infrastructure and signposting pathways are equally important. NICE guidance supports brief PA advice and consideration of appropriate follow-up according to individual need [3]. In practice, the usefulness of a PA conversation depends partly on whether clinicians can offer patients realistic next steps. Community exercise programs, leisure services, social prescribing, rehabilitation services, and condition-specific opportunities vary geographically and can change over time. Expecting individual clinicians to maintain detailed knowledge of these options is unlikely to be sustainable. Primary care networks (PCNs) and integrated care systems could support clinicians by maintaining accessible, regularly updated directories of local PA opportunities and referral pathways.

Improving PA counseling in UK general practice therefore requires more than increasing clinician awareness. The emphasis should shift toward making the desired behavior easier within existing systems. PA prompts could be incorporated more consistently into relevant chronic disease and preventive care templates, accompanied by appropriate structured coding. Practices and PCNs could establish clear local signposting pathways, while concise training could help clinicians initiate PA discussions, use behavior change techniques, and agree on realistic goals within time-limited consultations. Where appropriate, local quality improvement initiatives or organizational objectives could also help ensure that PA promotion is not repeatedly displaced by more immediately measurable clinical tasks.

PA counseling remains a low-cost, high-value intervention that is underused in UK general practice. Current evidence suggests that clinicians recognize its importance, but structural, digital, and organizational barriers make consistent implementation difficult. Existing systems such as QOF-linked long-term condition templates demonstrate that structured digital workflows can successfully prompt and record aspects of preventive and chronic disease care. The next step is not necessarily to create more standalone guidance, but to integrate PA assessment, counseling, coding, and signposting more consistently into the workflows clinicians already use. Doing so could help move PA promotion from an opportunistic addition toward a more reliable component of routine primary care.
